# The Association Between Diabetes and the Outcome of COVID-19 Infection in Bethlehem, Palestine: A Case–Control Study

**DOI:** 10.1155/2024/1051658

**Published:** 2024-11-12

**Authors:** Rasmi Fayiz Abu-Helu, Mohammad Jumah Zeer, Ghaleb Mohammad Adwan

**Affiliations:** ^1^Department of Medical Laboratory Sciences, Faculty of Health Professions, Al-Quds University, Jerusalem, State of Palestine; ^2^School of Public Health, Al-Quds University, Jerusalem, State of Palestine; ^3^Department of Biology and Biotechnology, An-Najah National University, P.O. Box 7, Nablus, State of Palestine

**Keywords:** COVID-19, diabetes, mortality, Palestine, risk factor

## Abstract

The severity of COVID-19's outcomes has been positively correlated with an increased risk of respiratory failure and death, especially in patients with chronic illnesses. This case–control design study aims to examine the correlation in the Palestinian population in light of its impact on diabetic patients. The study was conducted from March 2020 to June 2021 on 417 patients admitted to the Palestinian National Center for Rehabilitation. Of them, 198 cases were tested positive for COVID-19 and had diabetes, whereas the remaining 219 were those who tested positive for COVID-19 but were not diagnosed with diabetes and acted as controls. Data from patient files were collected to address the study questions. Patients' ages ranged from 17 to 98 years, with a mean age of 58. Male participants represented 53.5% of the total. The results of the current study indicated that the case fatality rate (CFR) was 14.2% for all participants and 19.7% for patients with diabetes. In regard to patients' health conditions, 5.7% had cardiovascular diseases (CVD), 33.6% had hypertension, 6.5% had kidney diseases, and 47.4% had diabetes. According to the multivariate analysis, diabetic patients had a 1.63 times higher risk for COVID-19 infection compared to nondiabetic patients (adjustment of odds ratio of 1.63 and confidence interval [CI] = 0.354–1.856). This is the first study to investigate the relationship between the severity of COVID-19 outcomes in Palestine and the diabetes status, and the majority of its findings are consistent with other research studies that assessed the impact of COVID-19 on diabetic patients (with minor variations in the number and percentage). The study confirms that along with diabetes, age, hypertension, kidney diseases, shortness of breath, requirement of oxygen, D-dimer, C-reactive protein (CRP), kidney function test (KFT), and days of hospitalization were also associated with severe COVID-19 outcomes, whereas gender, CVDs, and liver function test (LFT) were not associated with severe COVID-19 outcomes.

## 1. Introduction

Coronavirus disease is an outbreak caused by severe acute respiratory syndrome coronavirus 2 [1]. Early in December 2019, the first case was recorded in Wuhan city, China. The World Health Organization officially declared COVID-19 as a pandemic in March 2020 [2]. Coronaviruses are enveloped single-stranded RNA viruses that belong to the *Coronaviridae* family. The four most significant structural proteins of SARS-CoV-2 are the spike (S) protein-encoding gene, the envelope (E) protein-encoding gene, the membrane (M) protein-encoding gene, and the nucleocapsid (N) protein-encoding gene [3].

A COVID-19 infection can cause hyperglycemia in individuals without diabetes because the virus binds to ACE2 on pancreatic cells, which can impair *β*-cell function and cause insulin inadequacy. This could result in the development of diabetes in previously undiagnosed patients and explain the need for insulin in severe situations [4].

Individuals who suffer from underlying medical disorders such as diabetes or high blood pressure are more vulnerable to COVID-19. Indeed, diabetes seems to be one of the most common comorbidities among COVID-19 patients. Diabetic individuals have been found to have a higher chance of severe illness, worse prognosis, and higher death rates among affected patients [5, 6]. In a Chinese cohort study including 258 consecutively hospitalized COVID patients, diabetes was confirmed associated with greater disease severity and a higher risk of mortality in patients with COVID-19 [7]. Another study showed that poor fasting glucose levels and diabetes at hospital admission were substantially linked to higher odds of unfavorable outcomes, such as mortality, admission to an intensive care unit (ICU), and requirement of mechanical ventilation in COVID-19 patients [8]. A retrospective observational research carried out in Greece reported an association between Type 2 diabetes and higher disease severity and mortality in extremely sick COVID-19 patients [9]. A Chinese retrospective cohort study aimed to investigate the characteristics and risk variables associated with COVID-19 severity in COVID-19 patients. According to this study, worse results were linked to individuals who were elderly and had chronic illnesses [10]. The severity of COVID-19 infection and the high mortality rate among diabetic patients, particularly in older ages, may be explained by the fact that chronic diseases are linked to cardiovascular diseases (CVD), hypertension, chronic respiratory diseases, obesity, and kidney diseases [4, 11, 12]. Yet another retrospective cross-sectional report carried out in the United Kingdom showed that hospitalized COVID-19 patients with diabetes had a longer duration of stay in the hospital when compared to nondiabetic patients. In addition, this study reported that younger COVID-19 patients with diabetes and patients with diabetic ketoacidosis were more likely to survive when compared to older patients and patients without diabetic ketoacidosis [13].

According to the Palestinian Ministry of Health (MoH), there were more than 700,000 confirmed COVID-positive cases throughout Palestine as of September 4, 2022. Of them, 62% of these cases were reported in the West Bank and East Jerusalem and 38% in the Gaza Strip. There have been 5704 deaths overall: 65% distributed in the West Bank and East Jerusalem and 35% in the Gaza Strip. Since this study is about COVID-19 patients in Bethlehem, the number of cases was 30,780 and 309 patients died and the percent of vaccine coverage is 75% [14]. The distribution of cases in Bethlehem by gender was 55% males and 45% females. As reported, the distribution of COVID-19 patients in Bethlehem according to age group was 17.4%, 16.3%, 15.7%, 13.3%, 13.2%, 10.5%, and 0.6% for age groups 20–29, 10–19, 30–39, 40–49, 0–9, 50–59, and 60–69 years, respectively. Based on the annual MoH report in 2021, there were 4420 new cases of diabetes in the West Bank. The incidence rate was 166 per 100,000 of the West Bank population; 2065 of them were males, and 2355 were females, and 12.9% of deaths in 2021 were due to complications from diabetes [15].

The growing number of reported cases globally has prompted scientists to investigate and identify risk factors for higher infection rates [3]. Numerous investigations revealed that the majority of COVID-19-infected diabetic patients had mild to severe symptoms; some of them required mechanical breathing and admission to an ICU and generally had a higher death rate than other patients [8, 16]. According to reports from COVID-19 centers and specialists, many patients with diabetes experienced greater problems than others during the pandemic in Palestine. Understanding the association between the severity of COVID-19 infection and diabetes might help to predict patient outcomes and develop effective strategies to prevent disease complications. In addition to understanding that association, this study aims to explore the association between various factors such as sociodemographics, health conditions, vital signs and symptoms on admission, laboratory findings, and the outcome of COVID-19. Furthermore, it seeks to determine the association between diabetes and the composite outcome of infection (described by the need for ICU admission, oxygen therapy, and mechanical ventilation). This study is the first of its kind and can pave the way for a contextual understanding of diabetes and its relationship to COVID-19 in Palestinian patients.

## 2. Materials and Methods

### 2.1. Study Setting

This study was conducted at the Palestinian National Center for Rehabilitation (PNCR), a COVID-19 center in Bethlehem, which is governed by the Palestinian MoH. Bethlehem governorate is located in the south of the West Bank between Hebron and Jerusalem. It covers an area of 575 km^2^ which includes five main cities, 70 towns, and three Palestinian refugee camps. It has a population of about 244,704 [17]. This center is considered the first Palestinian center that specializes in treating drug addiction and was established on August 4, 2018, under the guidance of the Korean government foundation. In 2020, this 32-bed center was converted for treating COVID-19 patients.

### 2.2. Study Design

#### 2.2.1. A Case–Control Study

A case–control study design was chosen for its ability to identify the various factors that are related to both the severity of the COVID-19 outcomes and diabetes. It is easy to use, inexpensive, and beneficial in examining a variety of exposure variables. However, it should be noted that case–control studies are unable to determine the causation of the associations and are vulnerable to biases that may arise during the process of selecting variables [18].

### 2.3. Sample Frame

Cases and controls were the selected PNCR attendees who tested positive for COVID-19 and were diagnosed with diabetes and without diabetes, respectively.

### 2.4. Sampling Method

Stratified random sampling was used to select samples from homogenous population groups. This method was chosen because it identifies the differences between groups in a population, increases accuracy and precision, and minimizes errors when selecting the sample [19]. Participants were selected based on positive COVID-19 results, which were obtained via real-time polymerase chain reaction (RT-PCR) testing. They were then divided into two groups (cases and controls) based on their eligibility for diabetic diagnosis criteria. They were

  Cases: All COVID-19 patients with diabetes who attended PNCR between March 2020 and June 2021 were approached and participated in the study.
  Control: All COVID-19 patients without diabetes who attended PNCR between March 2020 and June 2021 were approached and participated in the study.

### 2.5. Exclusion Criteria

The exclusion criteria include patients who were discharged against advice, readmitted, pregnant, transferred to another hospital to complete their treatment, COVID-19-vaccinated, nonhospitalized, or triaged. Any patient without fasting blood sugar (FBS) results on admission was also excluded from the study.

### 2.6. Sample Size

The online software tool “Epitool” (AUVEST, n.d.) was used to determine the sample size for this study. The following parameters were used in the calculation: the association between diabetes and COVID-19 has an 80% power with confidence intervals (CIs) (95%), the estimated OR is 2.0, and the case–control ratio is 1:1.

### 2.7. Study Tool

For every patient participating in the study, the following information was retrieved from their patient records: age, gender, chronic disease history, signs and symptoms on admission, laboratory findings, and outcome composite. No other diseases such as respiratory virus infections (including influenza and respiratory syncytial) were noticed or diagnosed in the patients infected with COVID-19 during this study period.

### 2.8. Data Collection

Pilot study: Several files' contents were examined to ensure that all data were available in the PNCR, and a data collection sheet was prepared for this purpose.

The following data were retrieved: general information (age, gender, and history of chronic disease), signs and symptoms (headache, cough, general weakness, dyspnea, and fever), vital signs on admission (temperature, heart rate, systolic, diastolic, and oxygen saturation rate [SpO_2_]), laboratory tests (FBS, hemoglobin, white blood cells [WBCs], liver function tests [LFTs] [ALT and AST], kidney function tests [KFTs] [blood urea nitrogen [BUN], serum creatinine, D-dimer, C-reactive protein [CRP], and lactate dehydrogenase [LDH]]), and outcome assessment (oxygen supplement, hospital ward, date of hospitalization, and patient outcomes).

Any of the following criteria were used to identify diabetic patients: a history of the disease, the use of antidiabetic medications, a fasting blood glucose level of at least 7.0 mmol/L (1 mmol/L = 18 mg/dL), or a glycosylated hemoglobin level of greater than 6.5% [20].

All patients diagnosed with COVID-19 were approached and invited to participate in the study. Controls were selected randomly from COVID-19 patients who attended PNCR. Data regarding smoking status, body mass index (BMI), diabetic medication, radiology results, the complication of COVID-19 infection, the result of HbA1c, troponin I, serum electrolyte, PT, and PTT tests were unavailable.

### 2.9. Statistical Analysis

The data were statistically analyzed using SPSS version 20.0. Descriptive statistics were used to demonstrate percentages and frequencies of categorical variables. The continuous variables were compared between cases and controls by an independent sample *T*-test. The chi-square test was used to examine the association for categorical variables between cases and controls, where *p* < 0.05 was considered a significant association. Multiple logistic regressions were used to calculate the odds ratio (OR), as well as 95% CIs.

## 3. Results

### 3.1. Descriptive Analysis

This study consists of 417 participants; 198 patients were diabetic (cases), whereas 219 of them were nondiabetic (controls). The results of this study showed that the mean age of patients was 58 years, ranging from 17 to 98 years, 53.5% of whom were male. Thus, the male:female ratio was 1.15:1. As for patient outcomes, 59 patients died, and the case fatality rate (CFR) was calculated as 14.1% for all participants. The CFR among diabetic patients was 19.7%, whereas the CFR among nondiabetic patients was 9.13%. In regard to patients' health conditions, 5.8% had CVDs, 33.6% had hypertension, and 6.5% had kidney diseases. Because this study is a case–control study, the prevalence of diabetes was 47.4%. The results are presented in Table 1.

### 3.2. Sociodemographic Characteristics of Participants

The results of the current research revealed that there is no significant association between age and COVID-19 outcomes among diabetic patients (*p*=0.704). On the other hand, there is a significant association between age and COVID-19 outcomes among nondiabetic patients (*p*=0.001). The results also demonstrated no significant association between gender and COVID-19 outcomes among diabetic patients (*p*=0.447) and nondiabetic patients (*p*=0.747). The association between age (Table 2a) and sex (Table 2b) and diabetes status and the COVID-19 results are displayed below.

### 3.3. Chronic Diseases

Among diabetic patients, 15 had CVDs, 95 had hypertension, and 20 had kidney diseases. In the nondiabetic patient group, 9 had CVDs, 45 had hypertension, and 7 had kidney diseases. The data revealed that there is no significant association between CVDs and COVID-19 outcomes among diabetic patients (*p*=0.181) and nondiabetic patients (*p*=0.585). However, there is a significant association between hypertension and COVID-19 outcomes among diabetic patients (*p*=0.001) and among nondiabetic patients (*p*=0.030). Moreover, there is a significant association between kidney diseases and COVID-19 outcomes among diabetic patients (*p*=0.001), whereas there is no significant association between kidney diseases and COVID-19 outcomes among nondiabetic patients (*p*=0.126). The association between a history of chronic diseases and diabetes status and COVID-19 outcomes is presented in Table 3.

### 3.4. Signs and Symptoms

The majority of diabetic patients suffered from shortness of breath (87.9%), cough (70.1%), general weakness (67.2%), and fever (44.4%). Nondiabetic patients suffered from general weakness (65.8%), shortness of breath (65.3%), cough (65.7%), and headache (25.6%). Statistical analyses indicate that there is a significant association between general weakness (*p*=0.010) and shortness of breath (*p*=0.041) with COVID-19 outcomes among diabetic patients, whereas there is no significant association between headache (*p*=0.273), cough (*p*=0.082), and fever (*p*=0.905) with COVID-19 outcomes among diabetic patients. In nondiabetic patients, there is also a significant association between shortness of breathing (*p*=0.003) with COVID-19 outcomes, but there is no significant association between headache (*p* value = 0.256), cough (*p*=0.199), general weakness (*p*=0.361), and fever (*p*=0.717) with COVID-19 outcomes. The associations between signs and symptoms upon admission and diabetes status and COVID-19 outcomes are presented in Table 4.

### 3.5. Vital Signs

Average vital signs among diabetic patients include a body temperature of 38.26°C ± 0.85°C, systolic blood pressure of 125.61 ± 17.148 mmHg, and diastolic blood pressure of 74.42 ± 9.9 mmHg, whereas the average among nondiabetic patients includes a body temperature of 38.13°C ± 0.857°C, systolic blood pressure of 118.98 ± 14.658 mmHg, and diastolic blood pressure of 73.676 ± 8.630 mmHg. While statistical analyses revealed significant differences between systolic blood pressure means among diabetic and nondiabetic patients (*T*-test = 4.225, *p*=0.001), the same is not true for body temperature and diastolic blood pressure values. There were no significant differences between means among patient temperature on admission (*p*=0.741) and the diastolic blood pressure value (*p*=0.173) across the two groups (Table 5a). The data showed that 31 diabetic patients and 34 nondiabetic patients had tachycardia. The statistical analyses showed that there was no significant association between the heart rate and the COVID-19 outcomes among diabetic patients (*p*=0.328), nor among nondiabetic patients (*p*=0.562) (Table 5b). In addition, the results indicate that the average SpO_2_ among diabetic patients was 86% ± 7.6%; among nondiabetic patients, it was slightly higher (88% ± 7.8%). As for its relation with COVID-19 outcomes, the association is significant among diabetic patients (*p*=0.0001) and among nondiabetic patients (*p*=0.001) (Table 5c).

### 3.6. Laboratory Findings

Individuals with diabetes had greater mean values for the FBS test (287.78 mg/dL), D-dimer (2639.2 ng/mL), and CRP (117 mg/L). There were also some increases in the average levels of D-dimer (1153 ng/mL) and CRP (63 mg/L) among individuals who did not have diabetes. Statistical analyses showed no significant difference between the diabetes status and hemoglobin level (*p*=0.314); however, an independent *T*-test indicated significant differences between the diabetes status and FBS (*p*=0.001), D-dimer (*p*=0.001), and CRP (*p*=0.001) (Table 6a). There was an increase in the average ALT (42 IU/L), serum creatinine (1.3 mg/dL), urea (61 mg/dL), and LDH (392 IU/L) in individuals with diabetes and an increase in the mean ALT (46 IU/L) and LDH (342 IU/L) in those without diabetes. In addition, using the chi-square test in the diabetic patient group revealed a significant association between COVID-19 outcomes and serum creatinine levels (*p*=0.0001), urea (*p*=0.0001), CPK (*p*=0.005), and LDH (*p*=0.019), but no significant association between the outcomes and WBC (*p*=0.118), ALT (*p*=0.705), and AST (*p*=0.123). As for the control group, there is a significant association between the patients' COVID-19 outcomes and WBC (*p*=0.011), serum creatinine levels (*p*=0.012), urea (*p*=0.0001), CPK (*p*=0.005), and LDH (*p*=0.030). There is no significant association between ALT (*p*=0.052) and AST (*p*=0.121) with COVID-19 outcomes (Table 6b).

### 3.7. The Composite Outcome of COVID-19

#### 3.7.1. Supplement of Oxygen

The results of the current study showed that 74.2% of diabetic patients required low-flow oxygen supply, 15.7% required high-flow oxygen, and 10.1% of patients required mechanical ventilation (intubation). In regard to the controls, 87.2% of nondiabetic patients required low-flow oxygen supply, 10.0% required high-flow oxygen, and 2.7% of patients required mechanical ventilation. The chi-square test demonstrated a significant association between the oxygen supply and COVID-19 outcomes among diabetic patients (*p*=0.0001) and among nondiabetic patients (*p*=0.0001) (Table 7a).

#### 3.7.2. Days of Hospitalization

Based on the data, it was shown that the mean number of days hospitalized among patients with diabetes was 10.97 ± 6.6 days, while the mean among nondiabetic patients was 8.4 ± 6.22 days. A significant association was found between days of hospitalization and the COVID-19 outcomes among patients with diabetes (*p*=0.001) and a stronger association (*p*=0.0001) among the nondiabetic group. Data are presented in Table 7b.

#### 3.7.3. Hospital Ward

According to the results of this study, 31.8% (63/198) and 16.0% (35/219) of diabetic patients and nondiabetic patients were admitted to ICU, respectively. In addition, these results showed that there is a significant association between the need for intensive care and COVID-19 outcomes among diabetic patients (*p*=0.0001) and among nondiabetic patients (*p*=0.0001). The data from the chi-square test are shown in Table 7c.

### 3.8. Multivariate Analysis

All of the significant variables in the univariate analyses (*p* < 0.05) were included in a multivariate analysis to compare diabetic and nondiabetic patients with COVID-19 outcomes in light of an adjustment of odds ratio (AOR) and CI. The result showed a significant difference between the two groups (*p*=0.015) and revealed that diabetic patients with COVID-19 infection were associated with 1.63-fold increased risk of mortality and severe COVID-19 infection in comparison with nondiabetic patients (AOR: 1.63, CI = 0.354–1.856).  Table 8 exhibits the data.

## 4. Discussion

### 4.1. Study Participant Characteristics

#### 4.1.1. Age

The average participant age in this study was 58 years. Two similar retrospective investigations conducted in China report a mean age of 55.6 and 64 years [10, 21]. In a prospective cohort research conducted in China, the mean age was 63 [22]. In Iran, the majority of patients in the retrospective analysis were between the ages of 50 and 60 [12] and 63.5 years in a retrospective cohort study [23]. Furthermore, the median age in a Mexican case–control study was 45 years [24]. In Saudi Arabia, a retrospective study's mean age was 55 years [25].

Most of the studies indicated that older age was associated with an increased risk of mechanical ventilation and death. The majority of elderly patients had at least one chronic disease (such as diabetes, hypertension, CVDs, or lung disorders) and a weak immune system, which encumbered their ability to follow the COVID-19 infection prevention recommendations. Likewise, in this study, there was a significant correlation between age and COVID-19 outcomes among patients without diabetes. However, there was no correlation in the diabetic patient group. Thus, age was considered a confounder variable.

#### 4.1.2. Gender

This study was composed of 53.5% males and 46.5% females, and the results showed no significant association between gender and COVID-19 outcomes among either group. Two retrospective investigations conducted in China showed that 48.4% and 59% were males [10, 21], and in a prospective cohort study in the same country, 52% of patients were males [22]. In addition, two retrospective cohort studies in Iran reported that 41.8% and 51.8% of patients were males [12, 23]. A study based in the United States showed 59.1% of patients were males [26], while two studies in Kuwait reported that 66.2% of patients in the cross-sectional study were males [27], and 90% of patients in the retrospective study were males [28]. All of these studies reported that males were associated with a higher mortality rate. In general, males had a higher prevalence of getting hospitalized, severe symptoms, and greater mortality rates when compared to females. This may be due to several factors, including sexual hormone differences. Testosterone hormone action in males is immune-suppressing as opposed to the activating action of estrogen hormone. Generally, females have a stronger response of the immune system against infection [27]. In addition, ACE2, which has been postulated to increase the risk of acute lung injury, is approximately three times higher in males than in females [11].

### 4.2. Chronic Disease Prevalence and Severity of COVID-19 Outcomes

In this study, the prevalence of kidney diseases and CVDs was (6.5%) and (5.8%), respectively. No significant association was apparent between kidney diseases or CVDs and COVID-19 outcomes among nondiabetic patients. On the other hand, hypertension, which was reported in 33.6% of the cases, and diabetes, which was diagnosed in 47.5%, were both significantly associated with the COVID-19 outcomes among diabetic patients. Conversely, among patients with diabetes, there is no statistically significant association among cardiovascular and COVID-19 outcomes. Following AOR, the multivariate analysis confirmed that people with diabetes had a 1.63-fold greater risk of acquiring COVID-19 infection in comparison with patients without diabetes.

Based on data from a retrospective study performed on COVID-19 patients in China, 14.2% of the patients had serious medical conditions and 37.3% of the patients had more than one chronic illness [10]. Data from another retrospective observational analysis of COVID-19 patients in China revealed that patients with diabetes had a greater death rate than individuals without diabetes [21]. In Iran, a retrospective study found that a high death rate was significantly associated with the presence of comorbid diseases in the patients, such as diabetes, hypertension, cardiovascular illness, or chronic respiratory diseases [12]. This is in accordance with data from an observational cohort investigation carried out in the United States, where the prevalence of chronic diseases was linked to an increased risk of death and mechanical ventilation. The conditions that were most prevalent were hypertension (46.7%), diabetes (27.8%), CVDs (18.6%), and kidney diseases (12.2%) [26]. Based on data from a prospective cohort study of COVID-19 patients in China, abnormal kidney function and severe COVID-19 outcomes have a significant association [22].

Similar findings were also found in a case–control research among COVID-19 patients in Mexico. The data revealed the prevalence of CVDs (5.9%), diabetes (29.3%), and hypertension (36.1%). Diabetes and hypertension had a higher OR for suffering from severe COVID-19 outcomes. In contrast, Denova-Gutiérrez et al. [24] reported no evidence of a significant association between CVDs and the COVID-19 outcomes. Furthermore, according to a report on COVID-19 patients in China, the CFR was 10.5% for patients with CVDs, 7.3% for those with diabetes, and 6% for those with hypertension [29]. In accordance with a retrospective study among patients with COVID-19 in Saudi Arabia, the presence of diabetes (68.3%) and hypertension (42.6%) had a higher mortality rate (20.5%) than for those without diabetes [25].

Furthermore, a retrospective observational study conducted in the United States with COVID-19 patients found an association between diabetes and a poor clinical outcome [30]. A case–control research done in the United States reported a significant association between poor COVID-19 outcomes and the prevalence of diabetic patients (39.6%) [31]. Data from a retrospective cohort study performed in Iran indicated that 54% of patients had hypertension and 43.7% had heart problems that were strongly associated with a high death rate [23]. Additionally, in a systematic study conducted in Italy, diabetes was linked to a significantly greater risk of death (OR: 3.21) and ICU admission (OR: 2.79) among COVID-19 patients [32]. In an analysis of data from a retrospective single-center study of patients in Kuwait, diabetes and the elevated death rate among COVID-19 patients had a significant association [28].

The majority of research found that chronic diseases were associated with an increase in the mortality rate. Chronic disease is a risk factor for a wide range of diseases, including COVID-19. Patients with chronic diseases were more likely to experience complications such as multiorgan failure, acute respiratory distress syndrome, pneumonia, sepsis, and fatal outcomes related to immunological deficiencies. High ACE 2 expression on epithelial cells in the kidney, blood vessels, lungs, and intestines may allow COVID-19 to enter cells and cause organ failure. Furthermore, the binding of COVID-19 virus to ACE 2 on pancreas cells can disrupt the activity of *β*-cells and lead to insulin insufficiency, potentially resulting in a diabetic diagnosis and explaining the necessity for insulin in critical instances [4].

### 4.3. Signs and Symptoms

The majority of diabetic patients in this study suffered from shortness of breath (87.9%), general weakness (67.2%), cough (70.1%), headache (30%), and fever (40.4%). In the nondiabetic patient group, 65.3% suffered from shortness of breath, 65.8% from general weakness, 65.7% from cough, 25% from headache, and 43.8% from fever. The data showed that there is a significant association between general weakness and shortness of breathing with COVID-19 outcomes among diabetic patients, but no significant association between headache, cough, and fever with COVID-19 outcomes among diabetic patients. Otherwise, in the nondiabetic patient group, there is a significant association between COVID-19 outcomes and shortness of breath, but not with headache, cough, general weakness, and fever. According to a Chinese retrospective cohort study on COVID-19 patients, fever (79.5%), exhaustion (31.4%), dyspnea (24.2%), and chest tightness (23.2%) were the most common symptoms [10]. The most common symptoms among patients with severe COVID-19 infection, based on another retrospective observational study performed also in China, included fever (89.6%), cough (69.9%), and shortness of breath (59.6%) [21]. In a cross-sectional study among 284 COVID-19 patients in Kuwait, common symptoms were fever (85.6%), shortness of breath (49.7%), cough (45%), and headache (40.1%) [27]. In addition, the majority of patients (75.2%) in a Saudi Arabian retrospective analysis had a fever followed by shortness of breath (72.8%) and cough (70.0%). A small percentage of patients experienced vomiting, nausea, and diarrhea [25]. Furthermore, the Iran study indicated that dyspnea (56.7%), cough (45.9%), and fever (37.4%) were the most common symptoms in diabetic patients at admission [23]. Only a small number of studies confirm an association between the types of symptoms and COVID-19 outcomes; the majority exhibit varying results. In any case, signs and symptoms were crucial in determining the patient's state and the extent of their COVID-19 infection, and the diverging results may be attributed to smaller sample sizes and other factors.

### 4.4. Vital Signs

Between the cases and controls, there was a significant difference between the systolic blood pressure and the blood oxygen rate means. The association between them and the COVID-19 outcomes was also significant. On the other hand, there were no significant differences between means among the two groups in regard to diastolic blood pressure values and patient temperature on admission. In addition, there was no significant association between the heart rate and the COVID-19 outcomes among diabetic patients, nor among nondiabetic patients. In a retrospective study among COVID-19 patients conducted in Saudi Arabia, the data showed that the mean oxygen saturation was 91.0 ± 8.7 [25].

### 4.5. Laboratory Findings

In the present study, the FBS test (287.87 mg/dL), D-dimer (2639.2 ng/mL), CRP (117 mg/L), ALT (42 IU/L), urea (61 mg/dL), LDH (392 IU/L), and serum creatinine (1.3 mg/dL) were all elevated in the diabetic patient group. In addition, among patients without diabetes, there is an elevation in the average of D-dimer (1153 ng/mL), CRP (63 mg/L), ALT (46 IU/L), and LDH (342 IU/L). Furthermore, there were significant differences in the levels of diabetes-related markers such as LDH, FBS, D-dimer, CRP, serum creatinine levels, urea, and CPK. However, no significant association was observed between WBC, ALT, and AST with the COVID-19 outcomes. There was a significant association between WBC, serum creatinine levels, urea, CPK, and LDH in nondiabetic patients although no significant association was noted between ALT and AST with COVID-19 outcomes.

Laboratory findings from a retrospective study of COVID-19 patients in China demonstrated a decrease in the albumin and lymphocyte count and an elevation in the WBC count, ALT, AST, LDH, CRP, and serum creatinine [10]. The majority of COVID-19 patients in a Chinese prospective cohort analysis exhibited increases in CRP, ESR, and LDH, whereas 44.9% of patients had proteinuria, 14.1% had a high level of creatinine, and 13.1% had an elevated BUN level [22]. Laboratory data for fatal cases upon admission in an Iranian retrospective cohort study demonstrated a rise in the serum creatinine, WBC count, CRP, CPK, and LDH, and a reduction in the lymphocyte count [23]. A Chinese retrospective observational study revealed elevated levels of WBC, CRP, ferritin, procalcitonin, D-dimer, and LDH in diabetic patients with severe COVID-19 infection [21]. Additionally, a retrospective observational study performed in the United States revealed that those with diabetes had greater concentrations of LDH and CRP [30]. The laboratory results from a retrospective study in Saudi Arabia showed that all severe patients had greater concentrations of CRP, ferritin, LDH, and AST than nonsevere cases [25]. The laboratory results showed raised levels of inflammatory markers, high coagulation tests, and KFTs in a retrospective single-center research carried out in Kuwait [28]. A high prothrombin time and high D-dimer are indicators of hypercoagulability, a disorder produced by lung damage due to COVID-19. Furthermore, elevated BUN, serum creatinine, and inflammatory markers such as CRP all indicate the possibility of COVID-19 infection-related kidney injury, and as cells degrade, LDH increases [33]. The results of the laboratory tests in this study agreed with the findings of the majority of earlier studies.

### 4.6. The Composite Outcome of COVID-19

According to this study, 74.2% of patients with diabetes needed low-flow oxygen, 15.7% needed high-flow oxygen, and 10.1% needed intubation. In contrast, 87.2% of patients without diabetes required low-flow oxygen, 10.0% required high-flow oxygen, and 2.7% required intubation. The results of statistical analysis indicated a significant association between the oxygen supply and COVID-19 outcomes in both groups. According to the results of this study, 31.8% of patients with diabetes and 16.1% without diabetes were admitted to the ICU. Furthermore, 5.5% of patients required high-flow oxygen, while 64.68% required low-flow oxygen, implying that 16.8% of patients required mechanical ventilation. Furthermore, 20% of patients spent a mean of 5 days in ICU following hospitalization.

The statistical analysis indicated a significant association between the hospital ward and COVID-19 outcomes in both cases and controls. As reported by Yan et al. [21], a retrospective observational study conducted in China among patients with severe COVID-19 infection found that 47.2% were admitted to the ICU, with an average stay of 13 days. Fried et al. [26] reported on an observational cohort study conducted in the United States that determined the average length of hospitalization for all patients was 7 days, from admission to discharge or death. This is the first study to compare the number of hospitalized days and the outcomes between diabetic and nondiabetic patients. Based on the findings, patients with diabetes spent an average of 10.97 ± 6.6 days in the hospital, while people without diabetes spent an average of 8.4 ± 6.22 days there. Statistical analysis revealed a strong link between hospitalizations and COVID-19 results among both groups.

Hyperglycemia's effects on the immune system's ability to defend against infections, namely, on phagocytosis and WBC chemotaxis, have been linked to diabetes-related complications such as kidney diseases, heart diseases, and hypertension. These diseases have been connected to an increase in hospitalization days, the need for mechanical ventilation, and a high death rate [33]. The results of this study were comparable with those of most others based on the COVID-19 outcome composite.

The COVID-19 pandemic and the use of nonpharmaceutical interventions in public health since 2020 have had a significant impact on people's lives, communities, and governments. There have been noticeable alterations to the usual seasonal circulation patterns of common respiratory virus infections, such as those caused by the influenza virus and respiratory syncytial virus, since nonpharmaceutical interventions were created to reduce respiratory virus transmission. Other unintended consequences of the decline in community respiratory virus activity include a corresponding drop in the incidence of invasive *Streptococcus pneumoniae* infections [34].

## 5. Conclusion

This is the first study to investigate the relationship between the severity of COVID-19 outcomes and diabetes status in Palestine. To address the study question, a case–control study was conducted. The majority of the findings are consistent with other research studies that examine the impact of COVID-19 on diabetic patients, with minor variations in the number and percentage. Moreover, it confirms that diabetes is a risk factor that is linked to severe COVID-19 outcomes and a high mortality rate. Severe COVID-19 outcomes were also linked to age, hypertension, kidney diseases, shortness of breath, oxygen requirement, D-dimer, CRP, LDH, KFT, and hospitalized days; however, LFT, gender, and CVDs were not associated with the same aftereffect.

## Figures and Tables

**Table 1:** Descriptive sociodemographic characteristics of participants.

Parameters	Status	Female	Male	Total
		194 (46.5%)	223 (53.7%)	417
Age	Mean (min–max)	50 (17–50)	60 (30–98)	58 (17–98)

Diabetes	Infected	92 (90%)	106 (90%)	198 (47.5%)
	Uninfected	102 (10%)	117 (10%)	219 (52.5%)

Cardiovascular disease	Infected	15 (8.3%)	9 (4.2%)	24 (5.8%)
	Uninfected	179 (91.7%)	214 (95.8%)	393 (94.2%)

Hypertension	Infected	79 (68.6%)	61 (37.6%)	140 (33.6%)
	Uninfected	115 (41.4%)	162 (62.4%)	277 (66.4%)

Kidney disease	Infected	9 (4.8%)	18 (8.7%)	27 (6.5%)
	Uninfected	185 (95.2%)	205 (91.3%)	390 (93.5%)

Patient outcome	Death	26 (15.7%)	33 (17.3%)	59 (14.1%)
	Recovery	168 (84.3%)	190 (82.7%)	358 (85.9%)

**Table 2a:** Association between age and COVID-19 outcomes.

Diabetes status	Age	Patient outcome		Total	*p* (chi-square)
		Recovery	Death		
Diabetic	< 45	5	0	5	0.704
	46–55	36	8	44	
	56–65	50	13	63	
	> 66	68	18	86	
	Total	159	39	198	

Nondiabetic	< 45	66	1	67	**0.001** ∗
	46–55	64	2	68	
	56–65	28	6	38	
	> 66	41	11	58	
	Total	199	20	219	

^∗^Significant association.

**Table 2b:** Association between sex and COVID-19 outcomes.

Diabetes status	Sex	Patient outcome		Total	*p* (chi-square)
		Recovery	Death		
Diabetic	Male	83	23	106	0.447
	Female	76	16	92	
	Total	159	39	198	

Nondiabetic	Male	107	10	117	0.747
	Female	92	10	102	
	Total	199	20	219	

**Table 3:** Association between chronic diseases and COVID-19 outcomes.

Diabetes status			Patient outcome		Total	*p* (chi-square)
			Recovery	Death		
Diabetic	Presence of cardiovascular disease	Yes	10	5	15	0.181
		No	149	34	183	
		Total	159	39	198	
Nondiabetic		Yes	8	1	9	0.585
		No	191	19	210	
		Total	199	20	219	

Diabetic	Presence of hypertension	Yes	66	29	95	**0.001∗**
		No	93	10	103	
		Total	159	39	198	
Nondiabetic		Yes	37	8	45	**0.030∗**
		No	162	12	174	
		Total	199	20	219	

Diabetic	Presence of kidney disease	Yes	10	10	20	**0.001∗**
		No	149	29	178	
		Total	159	39	198	
Nondiabetic		Yes	5	2	7	0.126
		No	194	18	212	
		Total	199	20	219	

^∗^Significant association.

**Table 4:** Association between the presence of signs and symptoms upon admission and COVID-19 outcomes.

Diabetes status			Patient outcome		Total	Percentage (%)	*p* (chi-square)
			Recovery	Death			
Diabetic	Headache	Yes	51	9	60	30.0	0.273
		No	108	30	138		
		Total	159	39	198		
Nondiabetic		Yes	53	3	56	25.6	0.256
		No	146	17	163		
		Total	199	20	219		

Diabetic	Cough	Yes	108	32	140	70.1	0.082
		No	51	7	58		
		Total	159	39	198		
Nondiabetic		Yes	134	10	144	65.7	0.199
		No	65	10	75		
		Total	199	20	219		

Diabetic	General weakness	Yes	100	33	133	67.2	**0.010∗**
		No	59	6	65		
		Total	159	39	198		
Nondiabetic		Yes	129	15	144	65.8	0.361
		No	70	5	75		
		Total	199	20	219		

Diabetic	Shortness of breath	Yes	136	38	174	87.9	**0.041∗**
		No	23	1	24		
		Total	159	39	198		
Nondiabetic		Yes	124	19	143	65.3	**0.003∗**
		No	75	1	76		
		Total	199	20	219		

Diabetic	Fever	Yes	71	17	88	44.4	0.905
		No	88	22	110		
		Total	159	39	198		
Nondiabetic		Yes	88	8	96	43.8	0.717
		No	111	12	123		
		Total	199	20	219		

^∗^Significant association.

**Table 5a:** Association between vital signs on admission and COVID-19 outcomes.

Vital signs	Diabetes status	No.	Mean ± SD	*T*-test	*p*
Body temperature on admission	Diabetic	198	38.26 ± 0.850	−0.500	0.741
	Nondiabetic	219	38.13 ± 0.857		

Patients systolic on admission	Diabetic	198	125.61 ± 17.148	4.225	**0.001** ∗
	Nondiabetic	219	118.98 ± 14.658		

Patients diastolic on admission	Diabetic	198	74.42 ± 9.900	0.834	0.173
	Nondiabetic	219	73.676 ± 8.630		

Abbreviations: No., number of patients; SD, standard deviation.

^∗^Significant difference.

**Table 5b:** Association between the heart rate on admission and COVID-19 outcomes.

Diabetes status	Heart rate	Patient outcome		Total	*p* (chi-square)
		Recovery	Death		
Diabetic	60–100	137	30	167	0.328
	> 100	22	9	31	
	Total	159	39	198	
	60–100	169	16	185	

Nondiabetic	> 100	30	4	34	0.562
	Total	199	20	219	

**Table 5c:** Association between oxygen saturation rates (SpO_2_) on admission and COVID-19 outcomes.

Diabetes status	Mean (%)	Oxygen saturation rate	Patient outcome		Total	*p* (chi-square)
			Recovery	Death		
Diabetic	86 ± 7.6	> 95	16	1	17	**0.0001** ∗
		90–94	55	4	59	
		85–89	51	11	62	
		80–84	20	8	28	
		< 80	17	15	32	
		Total	159	39	198	
		> 95	44	0	44	
		90–94	63	4	67	

Nondiabetic	88 ± 7.8	85–89	60	6	66	**0.001** ∗
		80–84	16	3	19	
		< 80	16	7	23	
		Total	199	20	219	

^∗^Significant association.

**Table 6a:** Association between laboratory tests on admission with COVID-19 outcomes.

	Diabetes status	No.	Mean	SD	*T*-test	*p*
Glucose level (FBS)	Diabetic (min–max)	198	287.78 (132–569)	110.7	25.694	**0.001** ∗
	Nondiabetic (min–max)	219	95.11 (68–111)	7.13		

Hemoglobin	Diabetic	198	13.21	1.70	−1.008	0.314
	Nondiabetic	219	13.37	1.57		

D-dimer (normal range 0–500 ng/mL)	Diabetic	158	2639 ng/mL	3183	5.189	**0.001** ∗
	Nondiabetic	153	1153 ng/mL	1576		

CRP (normal range 0–6 mg/L)	Diabetic	145	117 mg/L	96.9	5.658	**0.001** ∗
	Nondiabetic	142	63 mg/L	59.3		

Abbreviations: No., number of patients; SD, standard deviation.

^∗^Significant difference.

**Table 6b:** Association between laboratory test results on admission and COVID-19 outcomes.

Diabetes status	Test	Result	Patient outcome		Total	*p* (chi-square)
			Recovery	Death		
Diabetic (mean = 10.4 × 10^9^/L)	WBC	< 4 × 10^9^/L	3	2	5	0.118
		4–11 × 10^9^/L	100	18	118	
		> 11 × 10^9^/L	56	19	75	
		Total	159	39	198	
Nondiabetic (mean = 9.4 × 10^9^/L)		< 4 × 10^9^/L	12	0	12	**0.011** ∗
		4–11 × 10^9^/L	138	9	147	
		> 11 × 10^9^/L	49	11	60	
		Total	199	20	219	

Diabetic (mean = 42 IU/L)	ALT	≤ 40 IU/L	107	25	132	0.705
		> 40 IU/L	52	14	66	
		Total	159	39	198	
Nondiabetic (mean = 46 IU/L)		≤ 40 IU/L	125	17	142	0.052
		> 40 IU/L	74	3	77	
		Total	199	20	219	

Diabetic (mean = 37 IU/L)	AST	≤ 40 IU/L	114	23	137	0.123
		> 40 IU/L	45	16	61	
		Total	159	39	198	
Nondiabetic (mean = 36 IU/L)		≤ 40 IU/L	151	12	163	0.121
		> 40 IU/L	48	8	56	
		Total	199	20	219	

Diabetic (mean = 61 mg/dL)	Urea	< 17 mg/dL	4	0	4	**0.0001** ∗
		17–45 mg/dL	88	8	96	
		> 45 mg/dL	67	31	98	
		Total	159	39	198	
Nondiabetic (mean = 38 mg/dL)		<17 mg/dL	19	1	20	**0.0001** ∗
		17–45 mg/dL	145	5	150	
		> 45 mg/dL	35	14	49	
		Total	199	20	219	

Diabetic (mean = 392 IU/L)	LDH	< 140 IU/L	6	0	6	**0.019** ∗
		140–280 IU/L	58	7	65	
		> 280 IU/L	86	31	117	
		Total	150	38	188	
Nondiabetic (mean = 342 IU/L)		< 140 IU/L	12	0	12	**0.030** ∗
		140–280 IU/L	79	4	83	
		> 280 IU/L	89	16	105	
		Total	180	20	200	

Diabetic (mean = 131 IU/L)	CPK	≤ 198 IU/L	130	26	156	**0.002** ∗
		> 198 IU/L	17	12	29	
		Total	147	38	185	
Nondiabetic (mean = 104 IU/L)		≤ 198 IU/L	154	13	167	**0.005** ∗
		> 198 IU/L	21	7	28	
		Total	175	20	195	

Diabetic (mean = 1.3 mg/dL)	Creatinine	< 1.2 mg/dL	128	18	146	**0.0001** ∗
		≥ 1.2 mg/dL	31	21	52	
		Total	159	39	198	
		< 1.2 mg/dL	178	14	192	**0.012** ∗
Nondiabetic (mean = 0.97 mg/dL)		≥ 1.2 mg/dL	21	6	27	
		Total	199	20	219	

^∗^Significant association.

**Table 7a:** Association between the oxygen supply and COVID-19 outcomes.

Diabetes status	Oxygen requirement	Patient outcome		Total (%)	*p* (chi-square)
		Recovery	Death		
Diabetic	Low flow	141	6	147 (74.2%)	**0.0001** ∗
	High flow	17	14	31 (15.7%)	
	Intubation	1	19	20 (10.1%)	
	Total	159	39	198 (100%)	

Nondiabetic	Low flow	188	3	191 (87.2%)	**0.0001** ∗
	High flow	11	11	22 (10%)	
	Intubation	0	6	6 (2.7%)	
	Total	199	20	219 (100%)	

^∗^Significant association.

**Table 7b:** Association between days of hospitalization and COVID-19 outcomes.

Diabetes status	Mean days of hospitalization	Days of hospitalization	Patient outcome		Total	*p* (chi-square)
			Recovery	Death		
Diabetic	10.97 ± 6.6	≤ 7	55	11	66	**0.001** ∗
		8–14	76	12	88	
		15–21	20	6	26	
		≥ 22	8	10	18	
		Total	159	39	198	

Nondiabetic	8.4 ± 6.22	≤ 7	128	5	133	**0.0001** ∗
		8–14	55	8	63	
		15–21	11	4	15	
		≥ 22	5	3	8	
		Total	199	20	219	

^∗^Significant association.

**Table 7c:** Association between hospital wards and COVID-19 outcomes.

Diabetes status	Hospital ward	Patient outcome		Total (%)	*p*
		Recovery	Death		
Diabetic	Medical ward	133	2	135 (68.2%)	**0.0001** ∗
	ICU	26	37	63 (31.8%)	
	Total	159	39	198 (100%)	

Nondiabetic	Medical ward	184	0	184 (84%)	**0.0001** ∗
	ICU	15	20	35 (16%)	
	Total	199	20	219 (100%)	

^∗^Significant association.

**Table 8:** Multivariate analysis of the association between diabetes status and COVID-19 outcomes after adjustment of the odds ratio.

Diabetes status	Patient outcome		Total	*p*	AOR	95% CI min–max
	Recovery	Death				
Diabetic	159	39	198	**0.015** ∗	1.63	0.354–1.856
Nondiabetic	199	20	219			

^∗^Significant association.

## Data Availability

The data that support the findings of this study are available on request from the corresponding author.
